# Relationship between Air Pollutant Exposure and Gynecologic Cancer Risk

**DOI:** 10.3390/ijerph18105353

**Published:** 2021-05-18

**Authors:** Qiwei Yu, Liqiang Zhang, Kun Hou, Jingwen Li, Suhong Liu, Ke Huang, Yang Cheng

**Affiliations:** 1 State Key Laboratory of Remote Sensing Science, Faculty of Geographical Science, Beijing Normal University, Beijing 100875, China; yqwei1223@foxmail.com (Q.Y.); houkun156@163.com (K.H.); liush@bnu.edu.cn (S.L.); 201931051031@mail.bnu.edu.cn (K.H.); chengyang@bnu.edu.cn (Y.C.); 2 Guangxi Key Laboratory of Spatial Information and Geomatics, Guilin University of Technology, Guilin 541004, China; lijw@glut.edu.cn

**Keywords:** air pollutant exposure, gynecologic cancer risk, association

## Abstract

Exposure to air pollution has been suggested to be associated with an increased risk of women’s health disorders. However, it remains unknown to what extent changes in ambient air pollution affect gynecological cancer. In our case–control study, the logistic regression model was combined with the restricted cubic spline to examine the association of short-term exposure to air pollution with gynecological cancer events using the clinical data of 35,989 women in Beijing from December 2008 to December 2017. We assessed the women’s exposure to air pollutants using the monitor located nearest to each woman’s residence and working places, adjusting for age, occupation, ambient temperature, and ambient humidity. The adjusted odds ratios (ORs) were examined to evaluate gynecologic cancer risk in six time windows (Phase 1–Phase 6) of women’s exposure to air pollutants (PM_2.5_, CO, O_3_, and SO_2_) and the highest ORs were found in Phase 4 (240 days). Then, the higher adjusted ORs were found associated with the increased concentrations of each pollutant (PM_2.5_, CO, O_3_, and SO_2_) in Phase 4. For instance, the adjusted OR of gynecological cancer risk for a 1.0-mg m^−3^ increase in CO exposures was 1.010 (95% CI: 0.881–1.139) below 0.8 mg m^−3^, 1.032 (95% CI: 0.871–1.194) at 0.8–1.0 mg m^−3^, 1.059 (95% CI: 0.973–1.145) at 1.0–1.4 mg m^−3^, and 1.120 (95% CI: 0.993–1.246) above 1.4 mg m^−3^. The ORs calculated in different air pollution levels accessed us to identify the nonlinear association between women’s exposure to air pollutants (PM_2.5_, CO, O_3_, and SO_2_) and the gynecological cancer risk. This study supports that the gynecologic risks associated with air pollution should be considered in improved public health preventive measures and policymaking to minimize the dangerous effects of air pollution.

## 1. Introduction

Air pollution is a serious problem in mainland China. In the East China wide haze events in January 2013, the highest hourly concentration of PM_2.5_ exceeded 1000 μg/m^3^ in Beijing [1]. Pollution is the largest environmental cause of disease and death in the world today. Besides causing respiratory diseases [2], air pollution has been suggested to be associated with skin diseases [3,4], pregnancy abortion [5], and cardiovascular disease [6,7]. Moreover, prolonged exposures to hazardous air pollutants may result in cancers [8,9,10] and chronic diseases [11,12]. Concern is growing that exposures associated with air pollution might contribute to women’s health, which is one of the most significant public health issues and a global priority.

In light of the existing studies [13,14], gynecological diseases are threatening the health of women all around the world with more than 100,000 women dying every year. Gynecological tumors bring a heavy burden of disease in many countries [15,16], especially in developing and underdeveloped regions [17]. Over the past two decades, the mortality rates of gynecological cancer in China have been increasing and it has become a major health concern for women [18]. Considerable evidence has testified the certain association between ambient air pollution and gynecological tumors, including breast cancer [19,20], ovarian cancer [21], cervical cancer [22], and uterine fibroid [23]. Several studies [21,22] were only conducted in developed countries with good air quality, which were difficult to indicate the relationship between the gynecologic cancer risk and air pollution in developing countries due to the severe air pollution. A study [24] conducted in China’s Shandong province recently estimated the impacts of PM_10_, O_3_ [15], and SO_2_ [25,26] on the gynecologic cancer. The annual average or multi-year average concentration of air pollutants was utilized in previous experiments [19,27] to examine the association of pollutants and cancer. Some clinical evidence supported the suggestion that the exposure to air pollutants will bring about abnormal DNA methylation [28,29], estrogenic action [9], and inflammation from systemic circulation [28,30], whereas the specific mechanism between air pollutants and these diseases is still unknown.

The relationship and the mechanism need to be further assessed based on the accurate exposure estimate on different exposure levels by using reliable data. To guarantee the reliability and validation of the study, two aspects of data are indispensable: that is, strictly managed data of air pollution from the government and clinical data containing detailed patients’ information. For the former, the reliable and relatively complete air pollutant data was released in China after 2013 [31] and it gave us access to use different possible pollutants for quantitative analysis_._ For the latter, we collected the clinical records of 35,989 women in Beijing from December 2008 to December 2017. Following the previous studies [5,32,33], the measurements from air monitoring stations nearest to the patient’s residence and working place were used as the main estimate for the short-term exposure concentration of patients.

Based on the air pollution data and clinical data, we quantitatively assessed the relationship between the gynecological cancer risk and the short-term exposure (within one year) concentration of particulate matter with diameter below 2.5 μm (PM_2.5_), carbon monoxide (CO), ozone (O_3_) and sulfur dioxide (SO_2_), respectively. To ensure the robustness of the study results, a series of the following analysis were performed: (1) to identify the characteristics of exposure–response association within different time windows; (2) to obtain the shape of the nonlinear association between the risk of gynecologic cancer and air pollutant exposure through the restricted cubic spline analysis we adopted; and (3) to evaluate the exposure–response relationship of each air pollutant in different concentration ranges.

## 2. Methods

### 2.1. Data

#### 2.1.1. Air Pollution Data

Hourly measurements of air pollutants included PM_10_, PM_2.5_, SO_2_, CO, NO_2_, and O_3_ from 34 monitoring stations were established in 2013 by the Ministry of Ecology and Environment (MEE, formally the Ministry of Environmental Protection). Since the dataset before June 2014 contained a large number of missing values, we only used the data from June 2014 through December 2017 in this study. The NO_2_ measurements were not used here due to concerns about contamination by other nitrogen species [34] and we did not get data of other NO_x_ measurements. We excluded the PM10 data, which contained a large number of missing values throughout the years. Daily concentrations of pollutants were computed as the 24-h mean based on hourly measurements. To estimate pollution exposure, pollution measurement stations were selected based on the working and residential addresses of each mother [5].

To extend the PM_2.5_ data time period previous to June 2014, we made use of the long-term measurements taken by the US Embassy (http://www.stateair.net/web/historical/1/1.html; one site only; accessed on 12 July 2018). The US Embassy data were shown to be consistent with the MEE measurements [35]. For data at each MEE site from June 2014 to December 2017, we established a linear relationship with the US Embassy data on an hourly basis. Measurements at each MEE site were highly correlated with those at the US Embassy site [5]. This allowed us to apply the linear relationship to prior periods when there were no MEE measurements, as done in [5] and here. Using only data from June 2014 through 2017 suggested a similar association between air pollution exposures and gynecologic cancer risks, which supported our use of US Embassy data for earlier times [5].

#### 2.1.2. Meteorological Measurement Data

Three-hourly data over 2009–2017 for air temperature (in °C) and RH (in %) at two meters above the ground were taken from the meteorological measurement station near the southwestern Fourth Ring Road of Beijing. Data at this station are reported to the World Meteorological Organization and maintained at the United States National Oceanic and Atmospheric Administration National Centers for Environment Information (NOAA NCEI) (https://www.ncdc.noaa.gov/isd/data-access; accessed on 14 September 2020). Daily mean air temperature and RH were derived from three-hourly data. 

#### 2.1.3. Interpolation of Missing Air Pollutants and Meteorological Data

The air pollution and meteorological data contained missing values at certain times. To fill in the missing meteorological or air pollution data to accurately quantify the exposure level of each participating woman, we interpolated the missing values using the same interpolation methods as our previous study did [36]. 

#### 2.1.4. Women’s Clinical Data

We collected, processed, and selected clinical data as follows.

Collection of clinical data: We collected clinical data of 54,043 women with gynecological diseases (10,502 women was diagnosed with gynecological cancer) in Beijing, China. 

Data screening: Women were asked to provide the address they lived at the longest half a year before diagnosis. Women were excluded from the current study if there were no records of their addresses. We did not account for women’s smoking status, since most Chinese women do not smoke, especially when they feel physically sick.

Basic statistics of finally selected data: After all the aforementioned exclusions, data for a total of 35,989 women in Beijing from 2009 through 2017 were valid for analysis. The selected information includes age, occupation, diagnosis date, and whether the woman was diagnosed with cancer. 

Moreover, we divided the whole data into several subgroups based on the selected sociodemographic characteristics from the clinical data, namely age and occupation, which was also considered in some existing studies [37,38].

### 2.2. Assessment of 35,989 Patients’ Air Pollutant Exposure

**Figure 1:**
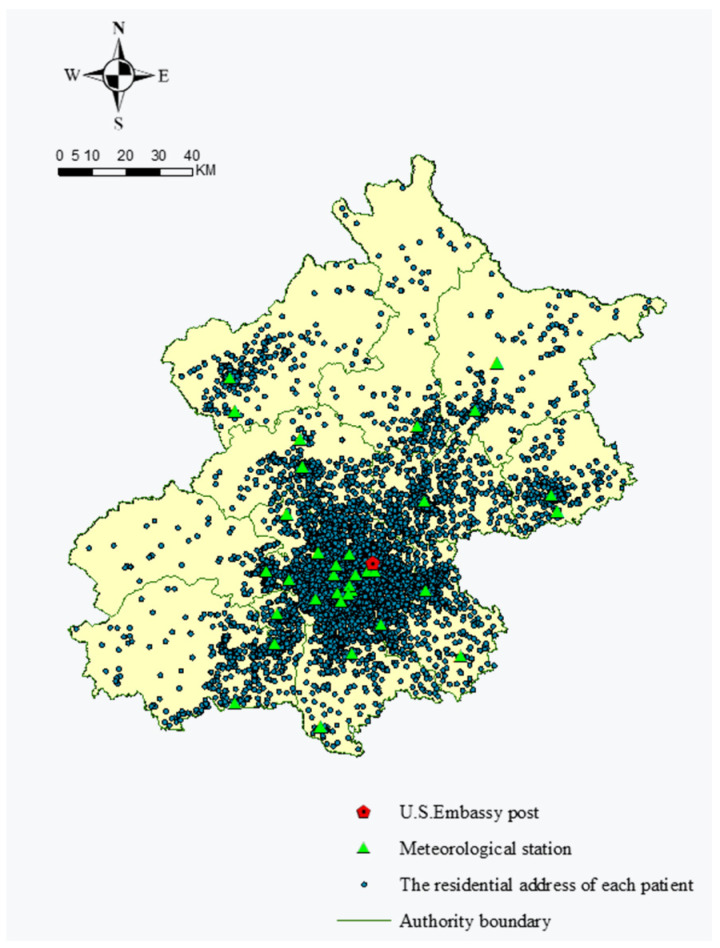
Spatial distribution of 35,989 patients’ residence and 34 air quality monitoring stations.

The studied women’s residential and working district addresses and air quality monitoring stations were geocoded to obtain their latitudes and longitudes. We estimated women’s exposure to air pollution by attributing representative concentrations provided by the air quality monitoring stations closest to the women residence and working place after geolocalization. The spatial distributions of 34 air quality monitoring stations and residential places of patients in Beijing are shown in Figure 1.

Approximately 86% participating women provided the working addresses. For each participating woman, the exposure concentration of *C*_d_ was computed as *C*_d_ = C_dw_/3 + 2C_dr_/3, where C_dw_ and C_dr_ denote air pollutant concentrations at the air monitoring stations closest to the participants’ working and residential addresses, respectively. The weights (1/3 and 2/3) approximately accounted for the times a participating woman spent at work and at home. For the other 14% of participating women who did not provide work addresses, we assumed that they did not go to work, and we only used their residence addresses to estimate the pollution exposure. 

To define the period of pollution exposure, we examined six time windows of women’s exposure to air pollution (Phase 1–Phase 6), each from 60, 120, 180, 240, 300, and 360 days before diagnosis of gynecologic cancer. We calculated the mean daily concentrations (i.e., the average of 24-h average across multiple days) of the pollutants in different periods (Phase 1, Phase 2, etc.) a woman was exposed. 

### 2.3. Variables Selection 

According to several previous studies [39,40], two-sided Student’s *t*-tests were usually performed to find the possible risk factors of gynecological cancer. Nonetheless, the normally distributed data are the critical condition for *t*-tests, so we needed to use the Kolmogorov–Smirnov test (K-S test) to identify the distribution of each factor among women. It is noted that the one-year average concentration of PM_2.5_, CO, O_3_, SO_2_, temperature, and relative humidity before the diagnosis date were usually used for the above tests [9]. The *p*-values of the K-S test were all less than 0.01, which shows that each mentioned factor did not follow the normal distribution. Therefore, we applied a Wilcoxon signed-rank test as an alternative to the Student’s *t*-test, which is a non-parametric statistical hypothesis test, and the distribution of data cannot be assumed to be normally distributed [41].

### 2.4. The Model

Firstly, we constructed a logistic regression model to evaluate the risk factors that influence gynecologic cancer. Potential confounding factors including age, occupation, ambient temperature, and ambient humidity were controlled in the final logistic regression model. Model results are reported in the form of odds ratios (ORs) [24,39] and their 95% confidence intervals (CIs). In addition, when associating each pollutant with gynecologic cancer, other pollutants were controlled in the logistic regression model [42]. The logistic regression model was formulated as:

(1) $$\ln\left(\frac{p}{1-p}\right)=\beta_{0}+\beta_{1}X_{1}+\beta_{2}X_{2}+\beta_{3}X_{3}+\beta_{4}X_{4}+\beta_{5}X_{5}+\beta_{6}X_{6}+\beta_{7}X_{7}+\beta_{8}X_{8}$$

where *p* represents the probability of gynecological cancer risk, *β*_0_ is a constant term. *β*_1_, *β*_2_, …, *β*_8_ represent the regression coefficients of independent variables *X*_1_, *X*_2_,..., *X*_8_. *X*_1_ is the ambient air temperature, *X_2_* is the relative humidity, *X*_3_ is the patients’ age, *X*_4_ is the patients’ occupation, *X*_5_ is the concentration of PM_2.5_, *X*_6_ is the concentration of CO, *X*_7_ is the concentration of O_3_, and *X*_8_ is the concentration of SO_2_. The OR value of each independent variable is $OR_{i}=exp\left(\beta_{i}\right)$. 

Then, in order to quantify the nonlinear relationship between the gynecological cancer risk and air pollutants, we combined logistic regression with restricted cubic spline as done in some previous studies [5,36]. In the restricted cubic spline, the value range of independent variables are divided, and the “node” is used to define the end of one interval and the beginning of the next interval. For example, [*a*, *b*] is the range of the independent variable and *k* nodes are used to divide the dataset into *k* + 1 intervals, i.e., $a=t_{0}<t_{1}<\ldots <t_{k-1}<t_{k}=b$. The spline function is a group of piecewise smoothly connected polynomials, and adoption of the spline function makes the final fitting curve smooth and continuous. Based on the regression spline, the restricted cubic spline has a constraint that the function is linear within the intervals of [$t_{0},t_{1}$] and [$t_{k-1},t_{k}$], and is represented by *RSC*(*X*):

(2) $$RCS(x,k)=\overset{k-1}{\underset{i=1}{\sum }}\beta_{i}S_{i}\left(X\right)$$

with:

(3) $$S_{1}\left(x\right)=x$$

(4) $$S_{2}\left(x\right)={\left(x-t_{1}\right)}^{3}{}_{+}-\frac{{\left(x-t_{k-1}\right)}^{3}{}_{+}\left(t_{k}-t_{1}\right)}{t_{k}-t_{k-1}}+\frac{{\left(x-t_{k}\right)}^{3}{}_{+}\left(t_{k-1}-t_{1}\right)}{t_{k}-t_{k-1}}$$

(5) $$S_{i}\left(x\right)={\left(x-t_{i-1}\right)}^{3}{}_{+}-\frac{{\left(x-t_{k-1}\right)}^{3}{}_{+}\left(t_{k}-t_{i-1}\right)}{t_{k}-t_{k-1}}+\frac{{\left(x-t_{k}\right)}^{3}{}_{+}\left(t_{k-1}-t_{i-1}\right)}{t_{k}-t_{k-1}}$$

(6) $${\left(x-t_{j}\right)}_{+}^{3}=\left\{\begin{array}{cc} {\left(x-t_{j}\right)}^{3} & \;\mathrm{if}\;x\geq t_{j}\\ 0 & \;\mathrm{else}\; \end{array}\right.$$

where *x* is the value of the continuous exposure variable *X*, *S_i_* is the spline function, *β_i_* is the estimator of *S_i_*, and k denotes the number of nodes. Four nodes representing the 25th, 50th, 75th, and 95th percentiles of PM_2.5_ concentration and three nodes representing the 25th, 50th, and 75th percentiles of CO, SO_2_, and O_3_ concentration were selected. The explanation for dividing 5 ranges for PM_2.5_ and 4 ranges for other pollutants is that the monitoring data shows that PM_2.5_ was the most serious pollutant in Beijing whose average concentration was 119.5 ± 13.8 μg m^−3^ in 2013 [43].

The final logistic regression model was combined with the restricted cubic splines to assess the exposure–response relationship, and the relationship between the exposure of each air pollutant and the risk of gynecological cancer was estimated through Equation (3).

(7) $$\ln\left(\frac{p}{1-p}\right)=\beta_{0}+\beta_{1}X_{1}+\cdots +\overset{k-1}{\underset{i=1}{\sum }}\beta_{i}S_{i}\left(X\right)$$

Since there was strong collinearity between PM_2.5_ and CO according to our previous study [5], we did not simultaneously control PM_2.5_ and CO as confounders in the model. That is, when controlling for PM_2.5_, we did not consider CO, and when controlling CO, we did not consider PM_2.5_. As we estimated the association between O_3_ and the gynecological cancer risk, we controlled for CO and SO_2_ but not PM_2.5_, given the strong correlation between PM_2.5_ and CO. Similarly, as we estimated the association between SO_2_ (CO) and the gynecological cancer risk, we controlled for CO (SO_2_) and O_3_ but not PM_2.5_.

## 3. Results

### 3.1. Linking Air Pollution Exposure to Gynecological Cancer Risks

All participants were grouped by age (6 groups), occupation (2 groups), temperature (5 groups), and relative humidity (5 groups). Table 1 shows the characteristics of gynecological non-cancer and cancer cases (age and occupation) and their environmental factors (like PM_2.5_, CO, SO_2_, O_3_, air temperature, and relative humidity). In addition, the Wilcoxon signed-rank test were performed to find the possible risk factors of gynecological cancer (See Section 2.3). The results showed that the differences of PM_2.5_, CO, O_3_, temperature, relative humidity, and occupation between the two groups (cancer and non-cancer) were statistically significant (*p* < 0.05), indicating that these factors are associated with the risk of gynecological cancer.

### 3.2. Associations between Gynecological Cancer Risk and Exposure to PM_2.5_, SO_2_, O_3_, and CO

#### 3.2.1. Correlations between Women’s Exposures in Different Time Periods

To determine if there was a difference on the risk of gynecological cancer in different exposure phases and which phase had the greatest risk of gynecological cancer, we considered six time windows of exposure (Phase 1–Phase 6), and the risks of PM_2.5_, CO, O_3_, and SO_2_ on gynecological cancer were quantified in each phase (see Section 2.2). 

**Figure 2:**
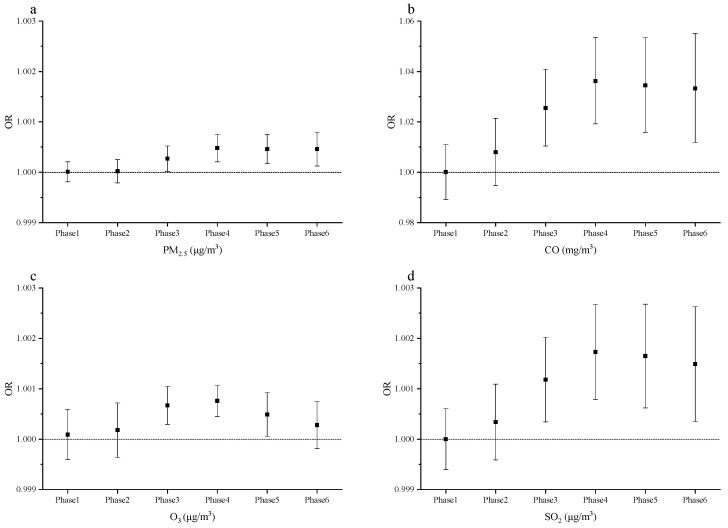
The relationship between the concentrations of four air pollutants and the gynecological cancer in six exposure phases. (**a**) The OR and 95% CI of gynecological cancer with a 1.0 μg/m^3^ increase in PM_2.5_ concentration; (**b**) the OR and 95% CI of gynecological cancer with a 1.0-mg/m^3^ increase in CO concentration; (**c**) the OR and 95% CI of gynecological cancer with a 1.0-μg/m^3^ increase in O_3_ concentration; (**d**) the OR and 95% CI of gynecological cancer with a 1.0-μg/m^3^ increase in SO_2_ concentration.

As demonstrated in Figure 2, the association between the risk of gynecological cancer and PM_2.5_, CO, O_3_, and SO_2_ exposures was statistically significant in each phase. Compared with other phases, the risk of PM_2.5_ exposures in Phase 4 (i.e., the period from 240 days to the participants with diagnosis of gynecological cancer) reaches the peak, and the risks of CO, O_3_, and SO_2_ exposures in Phase 4 were higher than the other phases. Thus, the average exposure concentration of each air pollutant in Phase 4 was used for further analysis in the following study.

#### 3.2.2. Impacts of Air Quality on Gynecological Cancer Risks

The logistic regression model was conducted to analyze four air pollutant exposures in Phase 4, and the relationship between the exposure concentration of four air pollutants and the OR value of gynecological cancer was obtained.

**Figure 3:**
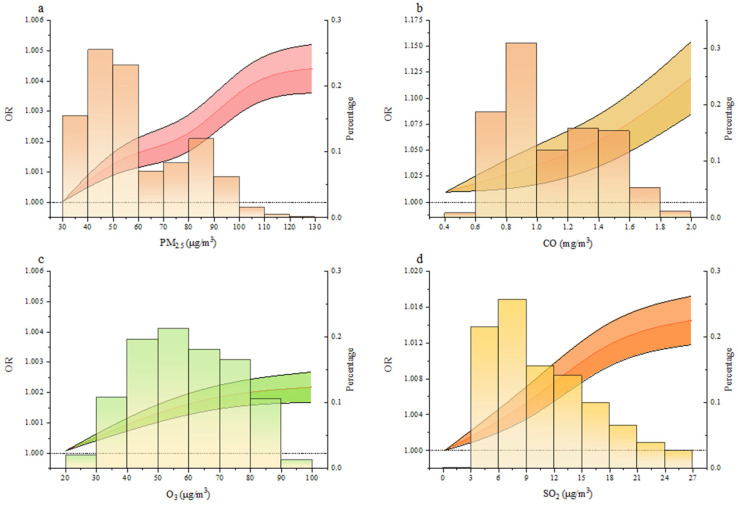
Exposure–response curve between air pollutant exposure and the risk of gynecological cancer. (**a**–**d**), Exposure–response curve of gynecological cancer risk with respect to PM_2.5_ (**a**), CO (**b**), O_3_ (**c**), and SO_2_ (**d**) exposure. The red line represents the OR value, and the black line denotes the 95% confidence intervals. The histogram shows the distribution of the corresponding pollutant concentration. Here we controlled confounders.

As shown in Figure 3, we combined the logistic regression model and the restricted cubic spline to describe the relationship between each of the air pollutants (PM_2.5_, SO_2_, CO, or O_3_) and the risk of gynecological cancer. 

**Figure 4:**
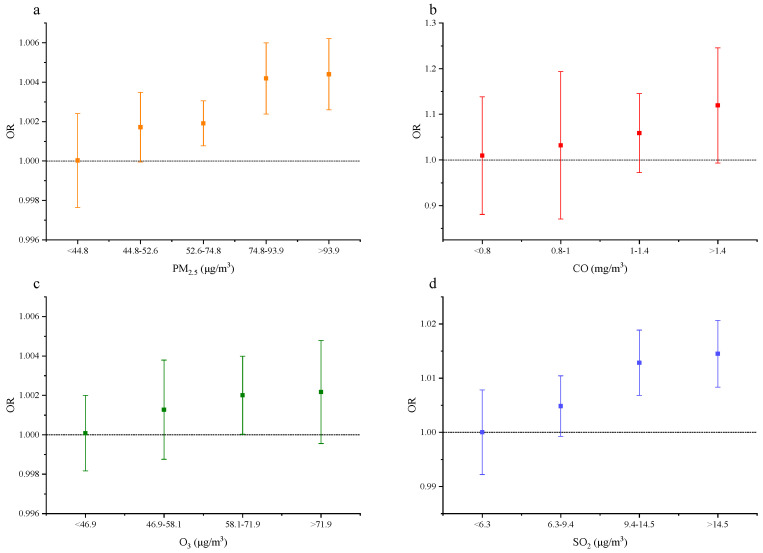
Influence of the four air pollutants on the risk of gynecological cancer in different ranges in Phase 4. (**a**–**d**), The OR of gynecological cancer with respect to PM_2.5_ (**a**), CO (**b**), O_3_ (**c**), and SO_2_ (**d**) exposure. The ORs of gynecological cancer with respect to PM_2.5_, CO, SO_2_, and O_3_ exposure. Here we controlled confounders.

As illustrated in Figure 4, for a 1.0-μg/m^3^ increase in PM_2.5_ exposures, the OR of gynecological cancer was 1.000 (95% CI, 0.998–1.002) for PM_2.5_ of <44.8 μg/m^3^, 1.002 (95% CI, 1.000–1.003) for PM_2.5_ of 44.8–52.6 μg/m^3^, 1.002 (95% CI, 1.001–1.003) for PM_2.5_ of 52.6–74.8 μg/m^3^, 1.004 (95% CI, 1.002–1.006) for PM_2.5_ of 74.8–93.9 μg/m^3^, and 1.004 (95% CI, 1.003–1.006) for PM_2.5_ > 93.9 μg/m^3^.

For a 1.0 mg/m^3^ increase in CO exposures, the OR of gynecological cancer is 1.010 (95% CI, 0.881–1.139) for CO <0.8 mg/m^3^, 1.032 (95% CI, 0.871–1.194) for CO of 0.8–1.0 mg/m^3^, 1.059 (95% CI, 0.973–1.145) for CO of 1.0–1.4 mg/m^3^, and 1.120 (95% CI, 0.993–1.246) for CO > 1.4 mg/m^3^.

For a 1.0-μg/m^3^ increase in O_3_ exposures, the OR of gynecological cancer was 1.000 (95% CI, 0.998–1.002) for O_3_ < 46.9 μg/m^3^, 1.001 (95% CI, 0.999–1.004) for O_3_ of 46.9–58.1 μg/m^3^, 1.002 (95% CI, 1.000–1.004) for O_3_ of 58.1–71.9 μg/m^3^, and 1.002 (95% CI, 1.000–1.005) for O_3_ > 71.9 μg/m^3^.

For a 1.0-μg/m^3^ increase in SO_2_ exposures, the OR of gynecological cancer was 1.000 (95% CI, 0.992–1.008) for SO_2_ < 6.3 μg/m^3^, 1.005 (95% CI, 0.999–1.010) for SO_2_ of 6.3–9.4 μg/m^3^, 1.013 (95% CI, 1.007–1.019) for SO_2_ of 9.4–14.5 μg/m^3^, and 1.015 (95% CI, 1.008–1.021) for SO_2_ > 14.5 μg/m^3^.

### 3.3. Associations between Air Pollution and Gynecological Cancer Risks by Sociodemographic Characteristics

Different sociodemographic status might play a crucial role in differentiating air pollution exposure among patients, which result in different risk for gynecological cancers. 

**Figure 5:**
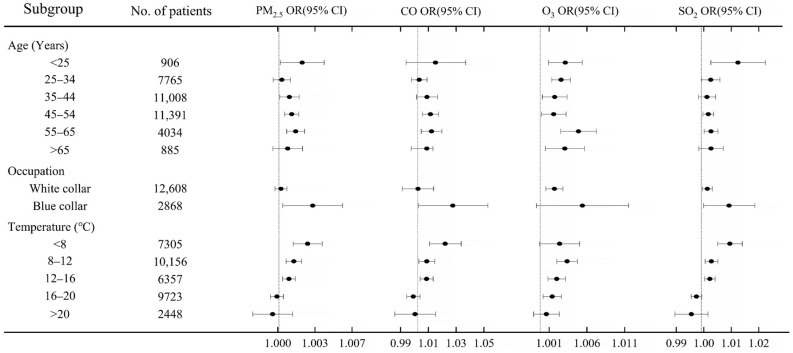
Relationship between air pollution and gynecological cancer with different social and demographic characteristics. The risks of four air pollutants on the risk of gynecological cancers is evaluated in each subgroup with PM_2.5_, O_3_, and SO_2_ increase by 1.0 μg/m^3^ and CO increase by 1.0 mg/m^3^.

As shown in Figure 5, when exposed to air pollution, the elderly patients (>65 years old) and blue-collar patients had a higher risk on gynecological cancer than their counterparts. 

### 3.4. Sensitivity Analysis

In the above sections, we assessed the influences of the four air pollutant exposures on the risk of gynecological cancer. Here we estimated the sole effect of each pollutant on gynecological cancer with versus without controlling for other air pollutants.

Considering that we did not have data of SO_2_, O_3_, and CO from December 2008 to May 2014, our sensitivity analysis only controlled for PM_2.5_ for this period. From June 2014 to December 2017, we took other air pollutants (PM_2.5_, SO_2_, O_3_, or CO) as confounders when the relationship between the exposure of one air pollutant and gynecological cancer. 

We found that the relationship between each pollutant and gynecological cancer, after controlling other pollutants, were very similar to the relationship without controlling other pollutants as shown in Table 2, Table 3, Table 4 and Table 5, suggesting that the influences of individual factors on gynecological cancer were largely independent.

## 4. Discussion

We quantitatively assessed the association between the air pollution exposures and gynecologic cancer risk. The findings indicate the increased risk of gynecologic cancer from exposure to higher concentrations of air pollutants. 

Some possible explanations to explain this association is as follows. When exposed to air pollution, the respiratory tract of people is certainly the primary damaged organs, but some studies confirmed that ultrafine particles from air pollutants can migrate through the blood to other organs and cause cancer [44]. Long-term exposure to hazardous environments dominated by air pollutants can induce oxidative stress reaction in cervical cancer cells, consequently damaging DNA and presenting similar symptoms to HPV infection [45], which is similar to the effect of smoking behaviors on the risk of gynecological tumors [22]. 

Air pollutant such as PM_2.5_ contain a variety of polycyclic aromatic hydrocarbons (PAHs) and its derivatives which are associated with genetic polymorphisms in the activation of certain carcinogens [25] and steroid hormone metabolism, thereby promoting the proliferation of cancer cells [46]. Substantial studies were in favor of the linkage between the PAHs and breast cancers [46], ovarian cancers [47], cervical epithelial tumors [48], uterine dysplasia [22], reproductive dysfunction, and pathological changes [49,50,51]. What is more, it should be emphasized that the PM_2.5_ can have the effect of estrogen [52], causing endocrine disorders. When inhaled, toxicants can bypass the liver metabolism and directly enter the systemic circulation [53]. Other air pollutants including CO, O_3_ were associated with the diseases concerning about fertility such as preeclampsia [54,55,56] and hypertensive disorders of pregnancy [57] in several existing studies. In addition, SO_2_ can lead to chromosomal aberrations, which can induce reproductive toxicity and carcinogenesis [5].

The findings about disparate exposure risks across different sociodemographic status support our further comparative analysis of these subgroup. In terms of age, it plays an essential role in cancer-related diseases [58,59,60]. The immunity of elderly patients was relatively weaker and might have some underlying diseases [61,62]. Therefore, even exposed to the same concentration of air pollution as young patients, it is likely to bring the elders with higher risk of gynecological cancers. As for occupation, the adverse health effects of air pollution varied depending on the socioeconomic status of patients. In China, compared with white-collar workers who usually work in offices, the female blue-collar workers have a lower socioeconomic status and are more frequently exposed to outdoor work [37], which poses a higher risk of air pollution-induced cancers. This finding is consistent with previous studies that high-income households are less susceptible to the adverse health impacts of exposure to hazardous air pollutants [38,63].

There are a few limitations in our studies. Firstly, there was no study on the effect of indoor air pollution on gynecological cancers due to the lack of indoor pollution data. As a result, we assumed that indoor and outdoor pollution were highly correlated [64]. Since the pollution data from the monitoring stations closest to the women’s residence (2/3 time) and workplace (1/3 time) cannot reflect individual exposure level, the risk of gynecological cancers may be underestimated or overestimated due to the frequency of the window opening and the source of indoor pollution [65]. Although we controlled confounders in the model, the effects of residual confounders were still hard to eliminate, such as smoking behavior. Last but not least, limited by the cognition of biological mechanisms on different gynecological cancer (e.g., breast cancer, ovarian cancer, and cervical cancer) may lead to the possibility of ecological fallacy. Hence, a massive crowd cohort study is necessary and gynecologic cancers in multiple sites on female body can be studied separately to obtain reliable public health evidence. Lastly, there is no consensus on the definition between long-term and short-term exposure to air pollution. Some studies consider the one year as the ‘long term’ [66], and others think it is the ‘short term’ [11,39]. We define the 1-year exposure as the short-term exposure, and our finding supports the association of the risk with short-term air pollution exposures is high. Nevertheless, we fail to conclude the association of the risk with long-term (multi-year) air pollution exposures, which is the limitation of our study.

China and other developing countries are still facing severe air pollution, although some governance policies have been taken in recent years [67]. Integrating the reduction of air pollution into future national policies to improve existing pollution problems is of irreplaceable importance for human health. Our quantitative spatial-temporal studies on the air pollution-induced risk of gynecological cancers contributed to a comprehensive understanding of the relationship between exposure to air pollution and gynecological cancers. Future work will utilize more types of data sources and consider more environmental confounding factors in an effort to characterize the impact of air pollution on human health as accurately as possible.

## Figures and Tables

**Table 1:** Results of Wilcoxon signed-rank tests in different characteristics and environmental factors.

Figure 2	Subgroup	Non-Cancer	Cancer	*p*-Values	PM_2.5_	CO	O_3_	SO_2_
Age (Years)	<25	830 (91.6%)	76 (8.4%)	0.011	0.039	0.040	0.005	0.003
	25–34	7192 (92.6%)	573 (7.4%)		0.041	0.009	0.051	0.002
	35–44	9830 (89.3%)	1178 (10.7%)		0.001	0.012	0.032	0.027
	45–54	9236 (81.1%)	2155 (18.9%)		0.000	0.042	0.002	0.017
	55–65	2294 (56.9%)	1740 (43.1%)		0.005	0.002	0.035	0.001
	>65	446 (50.4%)	439 (49.6%)		0.002	0.006	0.047	0.003
Occupation	White collar	11,180 (88.7%)	1428 (11.3%)	0.007	0.000	0.000	0.032	0.004
	Blue collar	2245 (78.3%)	623 (21.7%)		0.041	0.021	0.016	0.034
Temperature (°C)	<8	6010 (82.3%)	1295 (17.7%)	0.032	0.000	0.000	0.000	0.002
	8–12	8366 (82.4%)	1790 (17.6%)		0.031	0.034	0.029	0.047
	12–16	5328 (83.8%)	1029 (16.2%)		0.001	0.015	0.016	0.005
	16–20	8109 (83.4%)	1614 (16.6%)		0.017	0.020	0.048	0.006
	>20	2015 (82.3%)	433 (17.7%)		0.032	0.015	0.037	0.044
Relative Humidity (%)	<40	691 (85%)	122 (15%)	0.018	0.024	0.031	0.039	0.003
	40–50	9245 (87.3%)	1349 (12.7%)		0.012	0.024	0.021	0.025
	50–60	18,893 (86.2%)	3027 (13.8%)		0.008	0.007	0.019	0.020
	60–70	1832 (81.4%)	419 (18.6%)		0.037	0.002	0.001	0.036
	>70	360 (87.6%)	51 (12.4%)		0.005	0.002	0.043	0.010

Footnote: The air pollution levels in the table were calculated for the past 1 year from the diagnosis date.

**Table 2:** Influence of PM_2.5_ on gynecological cancers in Phase 4 with/without controlling other air pollutants.

PM_2.5_ (μg/m^3^)	ORs (Jun. 2014–Dec. 2017, Controlling for SO_2_ and O_3_)	ORs (Dec. 2008–Dec. 2017, without Controlling Other Pollutants)	ORs (Jun. 2014–Dec. 2017, without Controlling Other Pollutants)
<44.8	1.000 (0.998, 1.002)	1.000 (0.998, 1.002)	1.000 (0.998, 1.002)
44.8–52.6	1.002 (1.000, 1.003)	1.002 (1.000, 1.004)	1.002 (1.000, 1.004)
52.6–74.8	1.002 (1.001, 1.003)	1.004 (1.003, 1.005)	1.002 (1.001, 1.003)
74.8–93.9	1.004 (1.002, 1.006)	1.005 (1.003, 1.006)	1.003 (1.001, 1.005)
>93.9	1.004 (1.003, 1.006)	1.005 (1.003, 1.007)	1.004 (1.003, 1.006)

**Table 3:** Influence of SO_2_ on gynecological cancers in Phase 4 with/without control of other air pollutants.

SO_2_ (μg/m^3^)	ORs (Jun. 2014–Dec. 2017, Controlling for PM_2.5_, CO, and O_3_)	ORs (Jun. 2014–Dec. 2017, without Controlling Other Pollutants)
<6.3	1.000 (0.992, 1.008)	1.001 (0.993, 1.009)
6.3–9.4	1.005 (0.999, 1.010)	1.003 (0.997, 1.008)
9.4–14.5	1.013 (1.007, 1.019)	1.010 (1.004, 1.016)
>14.5	1.015 (1.008, 1.021)	1.011 (1.005, 1.018)

**Table 4:** Influence of CO on gynecological cancers in Phase 4 with/without control of other air pollutants.

CO (mg/m^3^)	ORs (Jun. 2014–Dec. 2017, Controlling for SO_2_ and O_3_)	ORs (Jun. 2014–Dec. 2017, without Controlling Other Pollutants)
<0.8	1.010 (0.881, 1.139)	1.003 (0.880, 1.125)
0.8–1	1.032 (0.871, 1.194)	1.055 (0.977, 1.133)
1–1.4	1.059 (0.973, 1.145)	1.071 (0.962, 1.180)
>1.4	1.120 (0.993, 1.246)	1.122 (0.944, 1.298)

**Table 5:** Influence of O_3_ on gynecological cancers in Phase 4 with/without control of other air pollutants.

O_3_ (μg/m^3^)	ORs (Jun. 2014–Dec. 2017, Controlling for PM_2.5_, SO_2_, and CO)	ORs (Jun. 2014–Dec. 2017, without Controlling Other Pollutants)
<46.9	1.000 (0.998, 1.002)	1.000 (0.998, 1.002)
46.9–58.1	1.001 (0.999, 1.004)	1.001 (0.999, 1.004)
58.1–71.9	1.002 (1.000, 1.004)	1.002 (1.000, 1.004)
>71.9	1.002 (1.000, 1.005)	1.002 (0.999, 1.004)

## Data Availability

The data that support the findings of this study are available from the corresponding author upon reasonable request.
